# Matched-Cohort DNA Microarray Diversity Analysis of Methicillin Sensitive and Methicillin Resistant *Staphylococcus aureus* Isolates from Hospital Admission Patients

**DOI:** 10.1371/journal.pone.0052487

**Published:** 2012-12-20

**Authors:** Ulla Ruffing, Ruslan Akulenko, Markus Bischoff, Volkhard Helms, Mathias Herrmann, Lutz von Müller

**Affiliations:** 1 Institute of Medical Microbiology and Hygiene, Saarland University Medical Center, Homburg/Saar, Germany; 2 Center for Bioinformatics, Saarland University, Saarbrücken, Germany; Beijing Institute of Microbiology and Epidemiology, China

## Abstract

As genotyping of *S. aureus* is important for epidemiologic research and for hygiene management, methods are required for standardized fast and easily applicable evaluation of closely related epidemic strains with high prevalence in hospitals. In this single centre matched control study we compared a new commercially available DNA microarray (IdentiBAC) with standard *spa*-typing for *S. aureus* genotyping. Included in the study was a subgroup of 46 MRSA and matched 46 MSSA nasal isolates of the Saarland University Medical Center collected during a state-wide admission prevalence screening. Microarray (MA) and also *spa-*typing could easily differentiate the genetically diverse MSSA group. However, due to the predominance of CC5/t003 in the MRSA group a sufficient subtyping required analysis of more complex genetic profiles as was shown here by the MA comprising a total number of 334 different hybridization probes. The genetic repertoire of the MRSA group was characterized by more virulence genes as compared to the MSSA group. The standard evaluation of MA results by the original software into CCs, *agr*-, SCC*mec*- and capsule-types was substituted in the present study by implementation of multivariate subtyping of closely related CC5 isolates using three different bioinformatic methods (splits graph, cluster dendrogram, and principal component analysis). Each method used was applicable for standardized and highly discriminative subtyping with high concordance. We propose that the identified *S. aureus* subtypes with characteristic virulence gene profiles are presumably associated also with virulence and pathogenicity *in vivo*; however, this remains to be analyzed in future studies. MA was superior to *spa*-typing for epidemiologic and presumably also provide functional respectively virulence associated characterization of *S. aureus* isolates. This is of specific importance for the hospital setting. In future, MA could become a new standard test for *S. aureus* typing in combination with multivariate bioinformatic analysis.

## Introduction

*Staphylococcus aureus* is a major human pathogen associated with invasive disease such as deep abscess formation, endocarditis, osteomyelitis, and sepsis [1]. The unabated global presence of methicillin-resistant *S. aureus* (MRSA) is a challenge for healthcare systems worldwide. Epidemic highly abundant MRSA strains of clonal origin have been characterized based on genetic profiles for healthcare associated (haMRSA), community associated (caMRSA) [2] and also for livestock associated infections (laMRSA) [3], [4]. Attempts have been made to associate *S. aureus* gene profiling [5]–[7] of clonal lineages with either ecological success [8] or clinical disease [9] yet, it remains to be determined which genetic traits render a given *S. aureus* clone to be clinical successful.

The focus to combat MRSA in hospitals must be on the reduction of MRSA transmission. Efficient transmission control, however, requires information on source and spread of nosocomial pathogens. Yet, this information is limited with regard to prevalent healthcare associated MRSA strains, as the typically clonal albeit regionally divergent phylogenetic traits of prevalent isolates [10] often preclude in-depth transmission pattern analyses. Moreover, the lack of routinely accessible information on the virulence gene equipment prevents any attempt for differentiated therapeutic or infection control approach as a function of pathogen equipment.

Genomic analysis of the variable X-region of the *S. aureus* protein A gene (*spa*) [11], [12] by single locus sequencing (*spa*-typing) has become very popular owed to its ease and standardized processing with easily applicable software tools and international databank functions [13], yet, the discriminatory power of *spa* analysis is limited in an epidemiological setting. It can be applied as a frontline tool for *S. aureus* typing; however, only in combination with additional discriminatory markers as e.g. SCC*mec* typing, lineage-specific genes or specific gene polymorphisms [12], [14]. Multilocus sequence typing (MLST) and to some extent also DNA macrorestriction appear to result in even smaller numbers of genotypes distinguishable. Multiple-locus variable-number tandem-repeat analysis (MLVA) [15]–[17] has provided added distinction even within similar genotypes, yet, MLVA includes multiple sequencing steps requiring expensive consumables and equipment optimized for this purpose. Complete genome analysis by next generation sequencing albeit successfully applied for outbreak analysis [18] will in the next future still remain an application for specialized laboratories. If applied to a specific cluster (e.g., the *t003* type) analysis of single nucleotide polymorphism (SNP) is able to further differentiate with a high discriminatory power, yet, in general each SNP probe is unique and restricted to respective clonal complexes [19].

Clonal lineage evolution in *S. aureus* has also been successfully analyzed by application of a microarray (MA) concept [6]. Moreover, a comprehensive approach through MA genomic hybridization has suggested that isolates from complicated infection may be differentiated from commensals as a result of virulence gene repertoire [20].

As a promising development towards ease-of-application, cost, and turnaround time, a commercial diagnostic DNA-based MA panel (Alere IdentiBAC® StaphyType Microarray [IdentiBAC MA]) has been developed for *S. aureus* genotyping [21]. The method is based on the comprehensive analysis of the *S. aureus* genome by hybridization to 334 different genetic probes [22], [23], and allows for highly reproducible simultaneous analysis of 174 genes dispersed over the complete *S. aureus* genome [24]–[26]. Genes analyzed can be grouped into lineage specific *S. aureus* genes, resistance and virulence genes [27]. As a result, *agr-*, capsule- and SCC*mec* typing as well as a highly accurate discrimination of *S. aureus* lineages is implemented [28], [29]. Crude IdentiBAC MA results are available in one working day and MA analysis has been already successfully applied for a broad collection of MRSA isolates [24], demonstrating 34 MRSA lineages and more than 100 different strains in human as well as veterinary isolates.

In this study, we have now employed IdentiBAC MA for a first time in a subgroup of MRSA and matched MSSA isolates collected during a large, state-wide admission prevalence screening in the State of Saarland (manuscript in preparation). Isolates of MSSA colonized patients matched for gender, age and previous hospital admissions were included as a control group of patients with similar predisposition and exposition to healthcare associated infections. MA analyses were complemented by *spa*-typing for independent lineage attribution, and results were subjected to advanced bioinformatic analysis. The study strived to address the following questions: i) What is the clonal lineage distribution of MSSA and MRSA isolates during a time and region-restricted hospital admission screening? ii) Can a difference in the accessory gene equipment of MRSA and MSSA hospital admission-associated isolates be observed? iii) Are there differences between bioinformatic models in respect to phylogenetic lineage delineation, and does bioinformatic analysis help to further differentiate between predominant clones indistinguishable by *spa*-typing and clonal complex (CC) attribution?

## Materials and Methods

### Patients and Clinical Isolates

Clinical isolates were collected in a 4 weeks interval during routine hospital entry screening from patients with nasal *S. aureus* colonization admitted to the Saarland University Medical Center. 46 MRSA isolates and 46 matched isolates of the MSSA colonized control group were included. Matched controls were selected according to gender, age (<70 vs. ≥70 years), previous hospitalizations in general and in the last 6 months (Table 1). Criteria were selected to match patients with a similar risk exposure for community and healthcare associated *S. aureus* contacts. The study was approved by the ethic commission of Saarland (registration # 127/10).

**Table 1:** Risk factors of MRSA and matched MSSA control group isolates.

Risk factors	MRSA, n (%)	MSSA, n (%)	p-value
Male	18 (39.13%)	18 (39.13%)	#
Female	28 (60.87%)	28 (60.87%)	#
<70 years	24 (52.17%)	24 (52.17%)	#
≥70 years	22 (47.83%)	22 (47.83%)	#
Hospitalisations <6 months	21 (45.65%)	21 (45.65%)	#
Inter-hospital transfer	5 (10.64%)	1 (2.17%)	ns
Previous MRSA colonization	3 (6.52%)	1 (2.17%)	ns
MRSA contacts	8 (17.39%)	4 (8.70%)	ns
Long-term care	11 (23.91%)	2 (4.26%)	0.014
Retirement home	3 (6.52%)	0 (0.00%)	ns
Diabetes mellitus	9 (19.57%)	8 (17.39%)	ns
Antibiotic therapy	21 (45.65%)	8 (17.39%)	0.007
Dialysis	3 (6.52%)	0 (0.00%)	ns
Medical devices	8 (17.39%)	0 (0.00%)	0.006
Skin lesions	6 (13.04%)	2 (4.26%)	ns

# statistical analysis was not performed for clinical criteria applied for selection of matched MSSA cases, ns =  not significant.

### *Spa*-typing

DNA of clinical isolates was prepared by boiling (95°C for 10 minutes) followed by amplification of the polymorphic X region of the protein A gene (*spa*) using standard primers *spa*-1113f (5′ TAA AGA CGA TCC TTC GGT GAG C 3′) and *spa*-1514r (5′ CAG CAG TAG TGC CGT TTG CTT 3′). Before sequencing (ITseq, Kaiserslautern, Germany) the PCR product was digested by Exo-SAP IT® (Affymetrix, Cleveland, United States) at 37°C (15 minutes), and the reaction was terminated at 80°C (15 minutes). Sequences were assigned into *spa*-types using the Ridom StaphType software version 2.1.1 and BURP algorithm (Ridom GmbH, Münster, Germany), as described previously [30].

### DNA Microarray-based Genotyping

DNA extraction and hybridization to the IdentiBAC MA (Alere Technologies GmbH, Jena, Germany) was performed as described in the manufacturer’s instructions [21], [27]. In brief, genomic DNA was purified using the cell lysis components of the assay in combination with DNeasy blood and Tissue kit (Qiagen, Hilden, Germany). The test principal is based on a linear multiplex primer elongation using one primer for every single target and DNA labeling by incorporation of biotin-16-dUTP. Following DNA hybridization, microarray probes were washed, then horseradish-peroxidase-streptavidin precipitation reaction was performed resulting in visible grey spots in case of a positive reaction. Spot signals were recorded, and automatically analyzed using the designated ArrayMate reader and the corresponding software (Iconoclust, Alere Technologies) [21]. As result, the MA readings of 334 target sequences corresponding to 174 distinct genes were classified into species markers, genes encoding virulence factors, microbial surface components recognizing adhesive matrix molecules (MSCRAMMS), antimicrobial resistance genes or SCC*mec-*, capsule- and *agr*- typing markers. As part of the IdentiBAC MA results in conjunction with the Iconoclust analysis, array profiles are attributed to a specific clonal complex (CC) and sequence type (ST) based on a proprietary algorithm provided by the manufacturer. Similarly, SCC*mec* types are attributed as a result of array signals obtained.

### Splits Graph Construction

A network tree was constructed by splits graph analysis (SplitsTree 4.11.3 software, www.splitstree.org) which was automatically linked to *spa*-typing results based on the computed export cost/distance matrix using the BURP algorithm of the Ridom StaphType software. The microarray results were imported directly into SplitsTree software 4.11.3 [31], and analyzed on default settings (characters transformation, uncorrected P; distance transformation, Neighbour-Net; and variance, ordinary least squares).

### Cluster Dendrogram Construction

Phylogenetic-like analysis of microarray hybridization pattern profiles was performed using R (version 2.13.1, http://www.r-project.org/) in conjunction with Bioconductor packages [32]. First, the data were preprocessed by removing all gene IDs containing ambiguous results. Afterwards, genes can only be present (´1′) or absent (´0′) in a particular sample. Next, the Euclidean distance matrix was computed to measure the similarity of gene hybridization profiles in different samples using the dist function in the software package “Stats” (R, version 2.13.1). Finally, a cluster dendrogram was constructed employing the hierarchical agglomerative clustering method and using by the hclust function in “Stats” that is based on Ward’s method [33], [34].

### Principal Component Analysis

As a multivariate analysis, principal component analysis (PCA) was carried out for *S. aureus* MA results to reduce the dimensionality of the MA data, and to identify groups of correlated variables. PCA characterizes the degree of variability (variance) observed among the detected genes. It combines the data for individual genes into so-called principal components (PCs) that are ordered according to the magnitude of variance observed in the data. Projecting the full data set onto the first few PC vectors showing the largest variance then allows a powerful reduction of data without loosing much information. The same preprocessed data was used as in the clustering analysis. PCs were computed by the R function prcomp in package “stats” with default parameters and the options retx =  TRUE, center =  TRUE and scale =  FALSE). By definition, the first principal component is the particular linear combination of gene hybridization profiles that contains the largest variation in the data. The second PC is the linear combination of the hybridization profiles that explains the largest variation after removing the first PC and so on. Here, only the first two PCs were considered for the present analysis.

### Statistics

Statistical evaluation was done by non-parametric tests using Fisheŕs exact test.

## Results

### Patients and Clinical Isolates

Patient characteristics were matched between the MRSA and the MSSA group for the selection criteria (sex, age, previous hospitalizations) whereas significant differences were found between groups for history of long-term care, previous antibiotic therapy, dialysis and the presence of medical devices (Table 1).

### *spa*-typing

**Figure 1:**
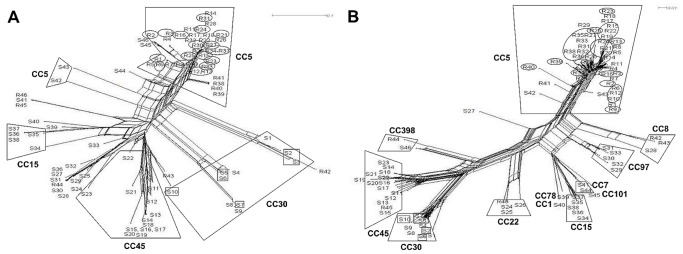
Diversity analysis of all MSSA (S1–S46) and MRSA (R1–R46) isolates by splits graph. (A) Splits graph constructed based on cost distance matrix produced by Ridom StaphType and (B) on default settings of the IdentiBAC microarray hybridization profiles of 334 genes and alleles. Clonal complexes (CC) as well as the most abundant *spa*-types t003 (circles) and t012 (quadrates) were highlighted.

The 46 MRSA isolates were assigned to 13 different *spa*-types (Table 2). The predominant MRSA *spa*-type was the epidemic strain *t003*, Rhine-Hesse (29, 63%). A higher diversity was uncovered among the 46 MSSA-isolates classified into 33 different *spa*-types with the most common MSSA *spa*-types being *t012* (6, 13%) and *t015* (5, 10.9%). For MSSA, *spa*-typing allowed for good discrimination of patient isolates which was shown here by splits graph analysis; however, the majority of MRSA isolates clustered into CC5/t003 which hampered sub-classification by *spa*-typing (Figure 1A).

**Table 2:** Differences of s*pa*-types and clonal complexes in MSSA and MRSA isolates.

Clonal complex	*Spa*-type	MSSA, n (%)	MRSA, n (%)
**CC1**	t8864	1 (2.17%)	0 (0%)
**CC5**	t003	0 (0%)	29 (63.04%)
	t504	0 (0%)	4 (8.70%)
	t010	0 (0%)	2 (4.35%)
	t002	1 (2.17%)	1 (2.17%)
	t045	0 (0%)	1 (2.17%)
	t481	0 (0%)	1 (2.17%)
	t493	1 (2.17%)	0 (0%)
	t887	0 (0%)	1 (2.17%)
	t1079	0 (0%)	1 (2.17%)
	t3195	0 (0%)	1 (2.17%)
**CC7**	t091	2 (4.35%)	0 (0%)
**CC8**	t008	1 (2.17%)	2 (4.35%)
**CC15**	t084	2 (4.35%)	0 (0%)
	t018	1 (2.17%)	0 (0%)
	t306	1 (2.17%)	0 (0%)
	t8786	1 (2.17%)	0 (0%)
**CC22**	t005	1 (2.17%)	0 (0%)
	t022	0 (0%)	1 (2.17%)
	t310	1 (2.17%)	0 (0%)
	t625	1 (2.17%)	0 (0%)
**CC30**	t012	6 (13.04%)	0 (0%)
	t019	1 (2.17%)	0 (0%)
	t273	1 (2.17%)	0 (0%)
	t584	1 (2.17%)	0 (0%)
	t8831	1 (2.17%)	0 (0%)
**CC45**	t015	5 (10.90%)	1 (2.17%)
	t026	1 (2.17%)	0 (0%)
	t040	1 (2.17%)	0 (0%)
	t050	1 (2.17%)	0 (0%)
	t073	1 (2.17%)	0 (0%)
	t339	1 (2.17%)	0 (0%)
	t620	1 (2.17%)	0 (0%)
	t1689	1 (2.17%)	0 (0%)
	t2239	1 (2.17%)	0 (0%)
**CC78**	t8863	1 (2.17%)	0 (0%)
**CC97**	t267	3 (6.62%)	0 (0%)
	t131	1 (2.17%)	0 (0%)
	t8831	1 (2.17%)	0 (0%)
**CC101**	t4044	1 (2.17%)	0 (0%)
**CC398**	t011	0 (0%)	1 (2.17%)
	t571	1 (2.17%)	0 (0%)
**unknown**	t078	1 (2.17%)	0 (0%)

### Clonal Complex Affiliation

Upon application of the original MA evaluation software (Iconoclust, Alere Technologies), isolates could be assigned to MLST clonal complexes (CCs) based on the hybridization profiles, except for two untypable MSSA isolates (S19, S2(Figure 1B). The MRSA isolates clustered into only five different CCs, while MA analysis of MSSA revealed twelve different CCs. MRSA isolates were dominated by CC5 (41, 89.1%) whereas the predominant MSSA types were found to be CC45 (12, 28.6%) and CC30 (10, 23.8%). Isolates of CC5, CC8, CC22, CC45 and CC398 were found both in the MRSA and the MSSA group, whereas CC30, CC15, CC97, CC7, CC1, CC78 and CC101 were present only in the MSSA group. CCs attributed to the MRSA group only were not found.

### Analysis of Gene Equipment

Microarray results of MRSA and MSSA isolates were analyzed for individual genes associated with e.g. antibiotic susceptibility, toxin production, adhesion and immune evasion. An overview of the most relevant genes in the investigated isolate cohort was provided for MRSA as compared to MSSA (Figure S1). Genes respectively gene components which were not detected in any cohort isolate were not displayed (*ermB, mefA, mph(C), vat(A), vat(B), vga, aphA3, sat, dfrS1, far1, cat, fexA, cfr, vanA/B/C, mercury resistance locus, qacA/C, seb,sef, she, seq, PVL, lukM, etB, edinA/D, splE, vwb, Q2YUB3*) as well as allelic variants (*vga, lukF, lukS, lukY, hlIII, aur, map, sdrC, sdrD, vwb, sasG, isaB, mprF, ImrP*). For more detailed analysis of selected gene profiles of individual isolates we refer to the supporting information (Table S1).

### *Agr*-typing

All CC5 isolates (n = 41, 89.13%) affiliated with *agr*II (accessory gene regulator type II). The remaining 5 MRSA isolates of CC8, CC22, CC45, CC398 (10.9%) as well as MSSA of CC7, CC22, CC45, CC97, CC101, CC398 (n = 26, 52.2%,) were associated with *agr*I, 12 MSSA isolates of CC1, CC30, CC78 with *agr*III (26.1%,) and 7 isolates of CC5 and CC15 with *agr*II (15.2%). The *agr* type of three MSSA isolates could not be determined using MA.

### SCC*mec* Typing

SCC*mec* types were identified based on hybridization patterns. Corresponding to the predominant clonal complex of the MRSA isolates all except four isolates of CC5 (37 of 41, 90.2%) comprised a SCC*mec*-cassette of type II. Isolates of the CC8 (n = 2), CC22 (n = 1), CC45 (n = 1) and one isolate of CC5 harbored the SCC*mec* type IV while the CC398 isolate were characterized by SCC*mec* type V. The SCC*mec* types of three isolates could not be determined by MA.

### Resistance Genes

MRSA isolates were defined and characterized by the detection of *mecA* in the SCC*mec* cassette. 39 MRSA isolates (84.8%) and also 29 (63.0%) MSSA isolates were positive for the β-lactamase operon (*blaZ,blaI,blaR)*. 43 (93.5%) MRSA yet only 20 (43.5%) MSSA isolates carried *fosB,* a putative marker for fosfomycin and bleomycin resistance (p<0.001); the detection of the *fosB* gene was limited to CC5, CC8, CC15, CC30, CC101. The macrolide, lincosamide and streptrogramin (MLS_B_) resistance gene e*rmA* was detected with significantly higher rates in the MRSA (41, 89.1%) as compared to the MSSA group (3, 6.5%) (p<0.001). Only one (2.2%) MSSA isolate was positive for *ermC*. The aminoglycoside resistance gene *aadD* was detected more frequently in MRSA (27, 58.7%) than in MSSA isolates (1, 2.2%) (p<0.001). Most isolates (84/92 [91.3%]) carried the unspecific efflux pump gene (*sdrM, formally tetEfflux*) which was equally distributed among MSSA and MRSA isolates. The tetracycline resistance gene *tet(K)* was detected in only one MRSA (2.2%) and two MSSA (4.3%) isolates, respectively.

### Virulence Genes

Panton-Valentine leukocidin (*pvl*) genes (*luk*F/S-PV) were not detected in the total study cohort. Only 9/46 (19.6%) MSSA isolates were *tst1* (toxic shock syndrome toxin) positive, most of them clustering into CC30 (8, 17.4%). The genetically linked leukocidin components (*lukD and lukE* as well as *lukS, lukF* and *hlgA*) were found more frequently in MRSA than in MSSA (p<0.001).

Among the haemolysin gene family, high abundance was detected among MRSA and MSSA for *hla, hlb, hld* and *hl*III, whereas differences between groups were detected for *hlb* (p<0.001).

The immune evasion gene cluster of *sak* (staphylokinase), *chp* (chemotaxis-inhibiting protein), or *scn* (staphylococcal complement inhibitor) was abundantly found both in the MRSA and the MSSA group.

Hybridization signals for exfoliative toxin *etA*, *etB*, *etD* and epidermal cell differentiation inhibitor edinA, edinB, edinC genes were detected only in a minority of strains.

The enterotoxin gene cluster (*egc* comprising *seg*, *sei*, *sem*, *sen*, *seo, seu)* was frequently identified both in MRSA (43/46, 93.5%) and MSSA (29/46, 63%) (p<0.001), yet, the gene cluster was restricted to isolates of CC5, CC22, CC30, CC45. Enterotoxin genes *sea, sed, sej* and *ser* were significantly more frequent in the MRSA group while all isolates were negative for *seb*, *sef*, *sek* and *seq* [35]. Interestingly, the 16 isolates of CC7, CC15, CC78, CC97, CC101 and CC398 (one MRSA and 15 MSSA) did not contain any hybridization signal for enterotoxin genes.

The serineprotease genes, *splA* and *slpB*, were predominantly found in the MRSA group (p<0.001), and this gene cluster was restricted to clonal complexes CC1, CC5, CC7, CC8, CC15 and CC97. The aureolysin gene (*aur*) was detected in 43 MRSA (93.5%) and 30 MSSA isolates (65.2%) (p<0.001). Other protease genes such as *sspA* (glutamylendopeptidase), *sspB* and *sspP* (staphopain B and A) were detected in the entirety of isolates tested. The ACME gene cluster, which had been brought to attention during analysis of caMRSA outbreak strains, was found in our population in the ST5-MRSA-II group (3, 6.5%).

Microbial surface components recognizing adhesive matrix molecule genes (MSCRAMM) comprising *cna* (collagen-binding adhesin), *sasG* (*S. aureus* surface protein G), *vwb* (van Willebrand factor binding protein) and *fib* (fibrinogen binding protein) are abundantly expressed, however, with higher proportions of *cna* positive isolates in the MSSA group, and higher rates of *fib*, *sas*G and *vwb* in the MRSA group. Other MSCRAMM genes such *bbp* (bone sialoprotein-binding protein), *clfA* (clumping factor gene A), *clfB* (clumping factor gene B), *ebh* (cell wall associated fibronectin-binding protein), *eno* (enolase binding protein), *ebpS* (cell surface elastin binding protein), *fnbA* (fibronectin-binding protein A) and *sdrC* (serine aspartate repeat fibrinogen binding protein) were found in the majority of strains without clear association to the methicillin resistance profiles.

As expected, the most obvious genetic differences in the highly abundant CC5 MRSA group (*bla*-operon, *aadD*, *sea*, *sed*, *sej*, *ser*, *hlb* and *chp*) were associated with altered mobile genetic elements.

More detailed characteristics of individual isolate in respect to *spa*-type, repeat succession, CC, SCC*mec*-type, *agr*-type, toxin profile, resistance profile, strain assignment and relation analyzed by hierarchical cluster dendrogram was shown in the supporting information (Figure S1).

### Microarray and *spa*-type Based Subclassification of CC5 Isolates

Most MRSA isolates were attributed to a genetic group of healthcare associated strains clustering into the CC5 (41, 89.1%). Except for two isolates of unidentified strain assignment, all isolates of CC5 referred to ST5-MRSA-II. This phylogenetically related and epidemiologically important CC5 was then selected for more detailed subtyping using MA hybridization as compared to classical *spa*-typing.

**Figure 2:**
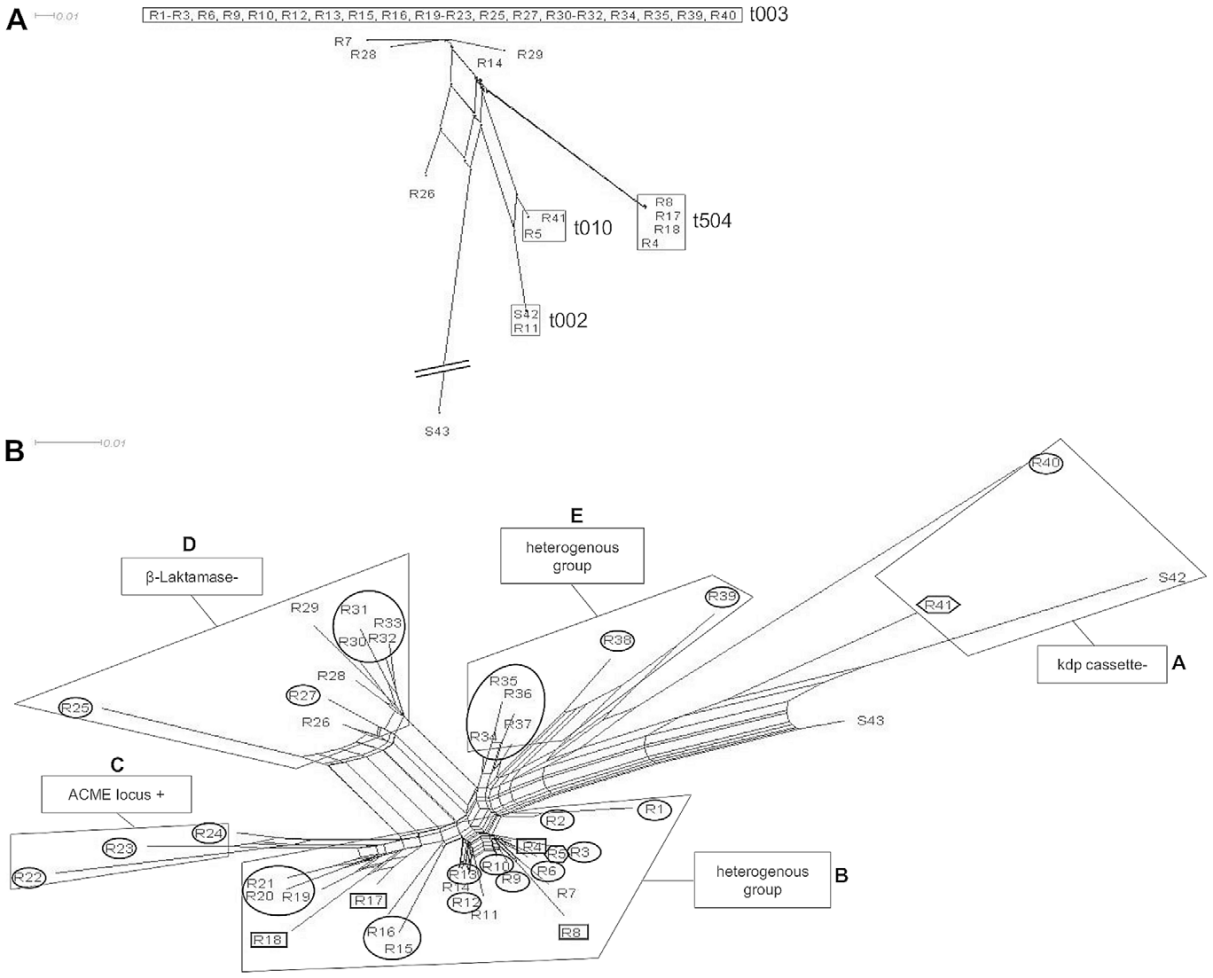
Subclassification analysis of 41 MRSA (R1–R41) and two MSSA (S42, S43) of CC5. (A) Splits graph based on cost distance matrix computed by Ridom StaphType software. (B) Splits graph based on MA hybridization profiles. Characteristic gene profiles for isolate cluster assignment were arbitrarily stated into group A-E. The most common MRSA *spa*-types t003 (circles), t504 (quadrates) and t010 (hexagons) were highlighed.

**Figure 3:**
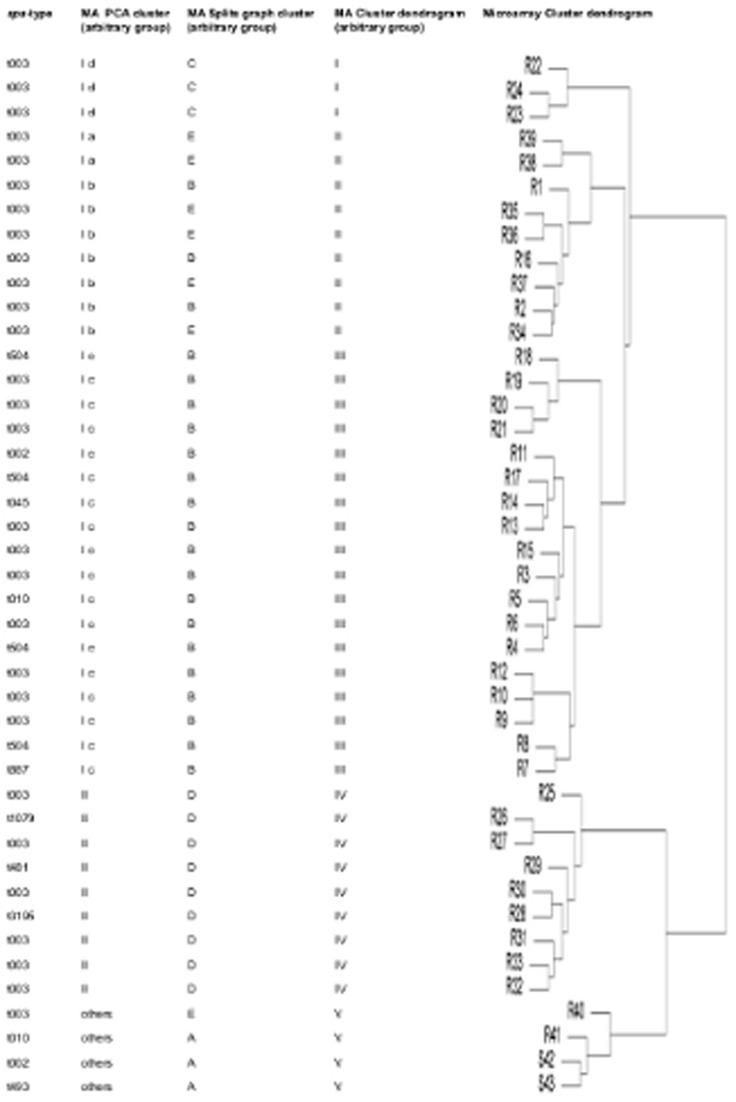
CC5 isolates (n = 43) characterized by *spa*-typing and comprehensive MA subgroup analysis using three different bioinformatic modes (principal component analyses, splits graph and cluster dendrogram).

**Figure 4:**
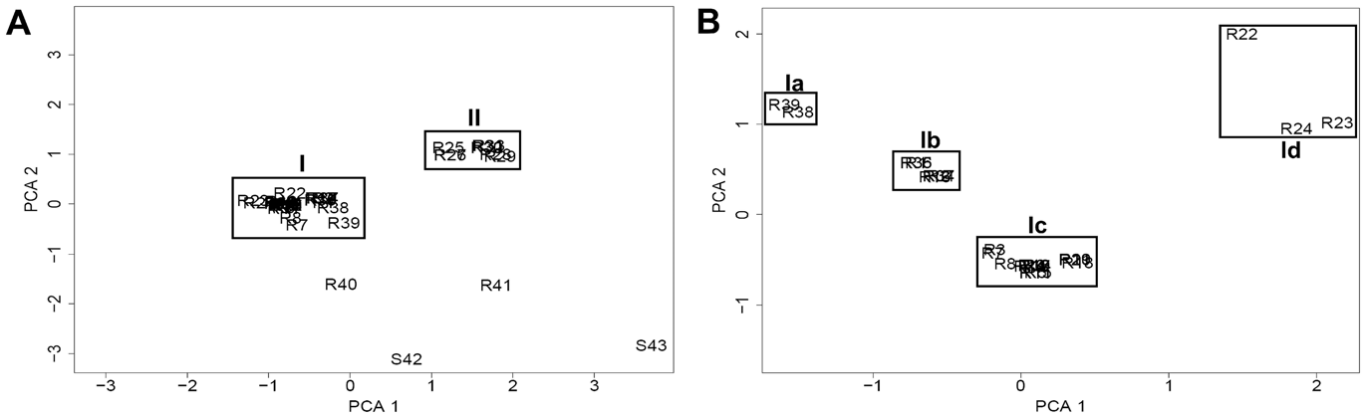
Principal component cluster analysis (PCA) of 41 MRSA (R1–R41) and two MSSA (S42, S43) of CC5. (A) Clustering of the 43 CC5 isolates by PCA as well as (B) subclustering of 30 MRSA CC5 cluster I isolates using a higher resolution PCA plot for in-depth identification of additional subgroups (Ia–Id).

A more detailed subtyping of *spa*-sequence data beyond the *spa*-type level was not possible as was demonstrated by splits graph distance matrix analysis (Figure 2A). Using the standard IdentiBAC MA software, subtyping of the MA results was not straight-forward. Instead, three alternative bioinformatics methods were found to be very helpful in subdividing genetically related strains by analysis of comprehensive genetic signatures determined by the MA. Results obtained by splits graph analysis (Figure 2B), cluster analysis using dendrograms (Figure 3), and principal component analysis (PCA) based on MA hybridization signals were evaluated (Figure 4). Splits graph of the MA results allowed subclassification of the 41 CC5 isolates into 5 different clusters (A-E), including subclassification of *spa*-type t003 and of both t010 isolates. Interestingly the t504 isolates with regional cumulation clustered exclusively into the subgroup B. Clusters A (*kdp* negative), C (ACME locus positive) and D (β-lactamase negative) were characterized by indicated specific genetic groups, whereas the genetic repertoire of cluster B and E was more heterogeneous. Cluster dendrogram of CC5 isolates revealed similar subclustering as compared to splits graph analysis except for few isolates (R1, R2, R11, R15, R16, R17). All CC5 cohort isolates were agrII and the majority of CC5 isolates with MRSA resistance profile were SCCmec type II positive strains of the Rhine-Hesse clone (95%).

Using PCA, 39 CC5 strains (90.9%) could be discriminated in two major clusters; additionally, four singleton isolates without clustering were found (9.1%) (Figure 4A). For more detailed information, the predominant cluster I (30 isolates) could be subdivided by focused PCA into four different subclusters (Ia-Id) (Figure 4B) resembling similar subtypes as compared to splits graph and cluster analysis (Figure 3).

## Discussion

In the present single centre study, the novel IdentiBAC MA platform was applied to the genotypic characterization of matched nasal methicillin sensitive and resistant *S. aureus* isolates collected upon patient admission to a tertiary care university hospital.

We could demonstrate that within the colonizing MSSA population tested, a large diversity of CCs was found in contrast to MRSA isolates with limited numbers of CCs and over-representation of CC5/*t003*. Low lineage diversity in the MRSA in contrast to the MSSA group was found very similarly also in clinical setting e.g. in cystic fibrosis patients [36]. Despite limited number of isolates the IdentiBAC MA revealed significant differences in the genetic repertoire of MRSA vs. MSSA isolates. Genetic differences were found to be distributed among various types of gene families including antimicrobial resistance genes, *agr* types and capsule type. In the present study the MRSA population was characterized by a significantly higher abundance of virulence genes attributed to the leukocidin, enterotoxin, haemolysin, protease and adhesion gene families, whereas only few single virulence genes (*tst, entL* and *cna*) were found more frequently in the MSSA group. Certainly, the genetic profile of the MRSA group was dominated by the genetic repertoire of one single epidemic MRSA clone (Rhine-Hesse); however, it may be also hypothesized that the Rhine-Hesse virulence gene repertoire was relevant for epidemic spreading of this successful epidemic MRSA clone. Of note, all isolates tested in this study were of commensal nature precluding association of virulence gene equipment with disease, yet, MA may become a regular diagnostic tool if specific clinical features could be associated with virulence gene patterns in subsequent studies.

In this study, it was demonstrated for the first time that evaluation of the raw IdentiBAC MA hybridization data by three independent bioinformatic methods allowed for in-depth phylogenetic MRSA isolate typing even beyond the prevalent CC5/*t003* MRSA genotype. Poor diversity of MRSA with predominance of CC5 isolates could be assumed as a limitation of this study; however, discrimination of these closely related strains is the most important challenge for analysis of healthcare-associated MRSA isolate cohorts obtained from geographically confined studies. In fact, it is the challenge for MA as a new alternative to established typing systems to overcome these limitations.

*Spa*-types and MA results were clustered into the same CCs; however, subclustering of the *spa*-types into STs [37] and also MA associated subtyps was not compelling. While genetic signatures of MA allow direct assignment to CCs and STs an assignment to *spa*-types cannot be achieved due to the heterogeneous genetic repertoire in the same *spa*-type. Single run IdentiBAC MA analysis in conjunction with appropriate software tools may now answer detailed questions both of epidemiologic as well as of infection control character.

Splits graph analysis by neighbor joining clustering, cluster dendrogram using hierarchical agglomerative clustering and also principal component analysis (PCA) formed very similar subgroups of the closely related CC5 isolates. In general, for more detailed strain assignment it has to be amended that a clearcut nomenclature discriminating strains and clones is still missing. In the present study, the CC5 subgroups characterized by a different lineage specific accessory gene repertoire were arbitrarily named group A-E. These predominant subgroups differed for specific gene families encoding β-lactamase resistance (*blaZ*/*blaI*/*blaR*) [38], the arginine catabolic mobile element (ACME) [39]–[41], the K^+^-transporting ATPase A-C chain, or the sensor histidine kinase, i.e. the *kdp* operon [42], [43]. ACME positive ST5-MRSA-II isolates have been identified before also in Hong Kong and USA (California) [24] which could be the base for new clone/substrain assignment by MA analysis. MRSA strains of the same CC can be attributed to characterized epidemic strains based on the presence/absence of characteristic genes. Thereby, the highly abundant toxic shock gene (*tst*) negative ST5-MRSA-II isolates were identified as Rhine-Hesse clone [44] whereas the CC8-MRSA-IV isolates were attributed to the Lyon clone [45], [46] due to their carriage of enterotoxin A (*sea*) with or without *sed*/*sej*/*ser*. The *tst* positive New-York Japan clone [47], [48] of ST5-MRSA-II was not detected in our population. By implementation of MA into routine diagnostics more detailed subtyping with elaborate techniques as e.g. whole genome sequencing [19] can be restricted to few closely related isolates with identical MA profiles clustering in the same genetic subgroup. Differences in characteristic gene families could result in altered metabolism and biologic activity. However, there is still limited evidence that genetically different subgroups may act differently according to *S. aureus* virulence *in vivo* [6], [49]–[51]. Additionally, also single nucleotide mutations beyond the resolution of the MA may influence the biologic behaviour of *S. aureus* strains which remains undetectable by MA [52]. Correlation between genotypic variants and clinical phenotype remains to be confirmed in future clinical studies.

While splits graph and cluster dendrogram evaluation are abundantly used for phylogenetic analysis [53], [54], PCA is a dimension reduction model becoming popular in recent years for genome-wide association studies [33], [34], [55], [56]. Thereby most of the original variability in the data can be retained without organizing them in a hierarchical format.

Comparing the three independent bioinformatic methods, a very similar sub-clustering of closely related CC5 isolates was demonstrated although each model may have its specific strengths for clinical application [55], [56]. The optimal choice between the three methods may indeed depend on the number of samples to be visualized and on the degree of diversity. For example, PCA enables a direct simple overview of an almost unlimited amount of isolates as shown here in the 2-dimensional graph. However, simple assignment of each point in the graph to the corresponding isolate is difficult in the case of densely overlapping samples. On the other hand, cluster dendrogram analysis reveals a more detailed isolate relationship with direct assignment of each isolate to the corresponding subgroup. Yet, this representation is most useful for sample sizes of less than a hundred. In the present case, splits graph analysis appeared to be most appropriate for diversity analysis during routine diagnostics due to ease-of-applicability, open-source software tools and direct assignment of each isolate to the branched subgroups in the 2-dimensional graph [31]. For future application of MA as an internationally accepted diagnostic tool it is important that a common standardized database-associated software tool is implemented independent of universally applicable bioinformatic tools investigated in the present study.

In conclusion, the present matched control study demonstrated a high genetic diversity for MSSA, either directly by *spa*-typing or by MA. However, differentiation of the predominant epidemic CC5 MRSA isolates was limited for *spa*-typing whereas detailed subtyping was achieved by bioinformatic-assisted MA analysis. The IdentiBAC MA could fulfil a number of criteria required for a new standard test for *S. aureus* typing including standardisation, ease of performance, low turn-around time (<24 hours), appropriate costs and superiority to established typing methods as was shown here for *spa*-typing. Based on the IdentiBAC MA concept, and as goal for the future development, standardized and easily applicable software tools based on the bioinformatic approaches with set highly differentiated strain assignment would then allow for comprehensive strain differentiation and global data exchange.

## Supporting Information

Figure S1 **Detailed characteristics of individual isolates (n = 92) in the cohort.** *Spa*-type, repeat succession, CC, SCC*mec*-type, *agr*-type, toxin and resistance profile, strain assignment and hierarchical clustering was of 46 MSSA (R1–R46) and 46 MSSA (S1–S46) was shown. Additionally also both major isolate groups were displayed (CC5 vs. others).
(XLS)

Table S1 **Genetic repertoire of MRSA and MSSA isolates.**
(DOCX)
