# Supplementary material for: Matched-Cohort DNA Microarray Diversity Analysis of Methicillin Sensitive and Methicillin Resistant Staphylococcus aureus Isolates from Hospital Admission Patients
Source: PLoS One. 2012 Dec 20;7(12):e52487. doi: 10.1371/journal.pone.0052487 (PMC3527544; doi:10.1371/journal.pone.0052487)
Supplement: Table S1 — Genetic repertoire of MRSA and MSSA isolates. (DOCX) [file pone.0052487.s002.docx]

| **Category** | **Description, corresponding target or type** | | **MRSA** | | **MSSA** | | **p- value** |
| --- | --- | --- | --- | --- | --- | --- | --- |
|  |  |  | **n** | **%** | **n** | **%** |  |
| Resistance | Gentamicin/tobramycin resistance gene | aacA-aphD | 1 | 2.17 | 1 | 2.17 | ns |
|  | Tobramycin resistance gene | aadD | 27 | 58.7 | 1 | 2.17 | <0.0001 |
|  | Beta-laktamase-operon | blaZ/ blaI/ blaR | 39 | 84.8 | 29 | 63.04 | ns |
|  | Trimethoprim resistance gene | dfrA | 1 | 2.17 | 0 | 0.00 | ns |
|  | Macrolide/ clindamycin resistance gene | ermA | 41 | 89.13 | 3 | 6.52 | ns |
|  | Macrolide/ clindamycin resistance gene | ermC | 0 | 0.00 | 1 | 2.17 | ns |
|  | Fosfomycin, Bleomycin | fosB | 43 | 93.48 | 20 | 43.48 | <0.0001 |
|  | Lincosamid resistance gene | lnu(A) | 0 | 0.00 | 1 | 2.17 | ns |
|  | Beta-lactam resistance | mecA | 46 | 100.00 | 0 | 0.00 | <0.0001 |
|  | Macrolid resistance gene | msr(A) | 0 | 0.00 | 1 | 2.17 | ns |
|  | Mupirocin resistance gene | mupA | 0 | 0.00 | 1 | 2.17 | ns |
|  | Hypothetical protein associated with fusidic acid resistance | Q6GD50 | 1 | 2.17 | 0 | 0.00 | ns |
|  | Putative transport protein (=tetEfflux) | sdrM | 42 | 91.30 | 42 | 91.30 | ns |
|  | Tetracycline resistance gene | tet(K) | 1 | 2.17 | 2 | 4.35 | ns |
|  | Tetracycline resistance gene | tet(M) | 1 | 2.17 | 0 | 0.00 | ns |
| *Agr*-type | Accessory gene regulator allele I | agrI | 5 | 10.87 | 24 | 52.17 | 0,00018 |
|  | Accessory gene regulator allele II | agrII | 41 | 89.13 | 7 | 15.22 | ns |
|  | Accessory gene regulator allele III | agrIII | 0 | 0.00 | 12 | 26.09 | 0.00021 |
| SCC*mec*-type | SCC*mec* - type II | II | 37 | 80.43 | 0 | 0.00 | <0.0001 |
|  | SCC*mec* - type IV | IV | 5 | 10.87 | 0 | 0.00 | ns |
|  | SCC*mec* - type V | V | 1 | 2.17 | 0 | 0.00 | ns |
| Toxins genes | Toxic Shock Syndrome Toxin 1 | tst1 | 0 | 0.00 | 9 | 19.57 | 0.0025 |
|  | Enterotoxin gene cluster | egc | 43 | 93.48 | 29 | 63.04 | 0.0007 |
|  | Enterotoxin A | sea | 26 | 56.52 | 9 | 19.57 | 0.0005 |
|  | Enterotoxin C | sec | 2 | 4.35 | 9 | 19.57 | 0.0499 |
|  | Enterotoxin D | sed | 32 | 69.57 | 2 | 4.35 | <0.0001 |
|  | Enterotoxin J | sej | 32 | 69.57 | 2 | 4.35 | <0.0001 |
|  | Enterotoxin K | sek | 0 | 0.00 | 0 | 0.00 | ns |
|  | Enterotoxin L | sel | 2 | 4.35 | 9 | 19.57 | 0.0499 |
|  | Enterotoxin R | ser | 31 | 67.39 | 2 | 4.35 | <0.0001 |
|  | Exfoliative Toxin serotype A | etA | 1 | 2.17 | 0 | 0.00 | ns |
|  | Exfoliative Toxin serotype D | etD | 1 | 2.17 | 0 | 0.00 | ns |
|  | Epidermal cell diff. inhibitor B | edinB | 0 | 0.00 | 1 | 2.17 | ns |
| Leukocidins | Haemolysin γ /Leukocidin, component A | hlgA | 44 | 95.65 | 39 | 84.78 | ns |
|  | Leukocidin D component | lukD | 43 | 93.48 | 19 | 41.30 | <0.0001 |
|  | Leukocidin E component | lukE | 42 | 91.30 | 15 | 32.61 | <0.0001 |
|  | Haemolysin γ /Leukocidin, component B | lukF | 45 | 97.83 | 44 | 95.65 | ns |
|  | Haemolysin γ /Leukocidin, component C | lukS | 44 | 95.65 | 13 | 28.26 | <0.0001 |
|  | Leukocidin/ Haemolysin toxin family protein | lukX | 42 | 91.30 | 40 | 86.96 | ns |
|  | Leukocidin | lukY | 46 | 100.00 | 46 | 100.00 | ns |
| Haemolysins | Putative membrane protein | hl | 46 | 100.00 | 43 | 93.48 | ns |
|  | Haemolysin α | hla | 42 | 91.30 | 43 | 93.48 | ns |
|  | Haemolysin β | hlb | 38 | 82.61 | 22 | 47.83 | 0.0009 |
|  | Haemolysine β without phage insertion | undisrupted hlb | 4 | 8.70 | 4 | 8.70 | ns |
|  | Haemolysin δ | hld | 46 | 100.00 | 46 | 100.00 | ns |
|  | Putative haemolysin III | hlIII | 45 | 97.83 | 43 | 93.48 | ns |
| Immune evasion | Chemotaxis inhibitor protein | chp | 33 | 71.74 | 31 | 67.39 | ns |
|  | Staphylokinase | sak | 42 | 91.30 | 36 | 78.26 | ns |
|  | Staphylococcal compl. inhibitor | scn | 42 | 91.30 | 42 | 91.30 | ns |
| ACME locus | Arginine catabolic mobile element | ACME | 3 | 6.52 | 0 | 0.00 | ns |
| Proteases | Aureolysin | aur | 43 | 93.48 | 16 | 34.78 | <0.0001 |
|  | Serineprotease A | splA | 43 | 93.48 | 18 | 39.13 | <0.0001 |
|  | Serineprotease B | splB | 43 | 93.48 | 19 | 41.30 | <0.0001 |
|  | Glutamylendopeptidase | sspA | 46 | 100.00 | 46 | 100.00 | ns |
|  | Staphopain B, protease | sspB | 46 | 100.00 | 46 | 100.00 | ns |
|  | Staphopain A,(staphylopain A) protease | sspP | 46 | 100.00 | 46 | 100.00 | ns |
| Caspule type | Capsule Type 5 | capsule 5 | 44 | 95.65 | 13 | 28.26 | <0.0001 |
|  | Capsule Type 8 | capsule 8 | 1 | 2.17 | 33 | 71.74 | <0.0001 |
| Biofilm | Intercellular adhesion protein A | icaA | 46 | 100.00 | 46 | 100.00 | ns |
|  | Intercellular adhesion protein C | icaC | 45 | 97.83 | 42 | 91.30 | ns |
|  | Biofilm PIA synthesis protein D | icaD | 46 | 100.00 | 45 | 97.83 | ns |
| Adhesion | Bone sialoprotein-binding protein | bbp-all | 42 | 91.30 | 42 | 91.30 | ns |
|  | Clumping factor A | clfA | 46 | 100.00 | 46 | 100.00 | ns |
|  | Clumping factor B | clfB | 46 | 100.00 | 43 | 93.48 | ns |
|  | Collagen-binding adhesin | cna | 3 | 6.52 | 28 | 60.87 | <0.0001 |
|  | Cell wall associated fibronectin-binding protein | ebh | 45 | 97.83 | 42 | 91.30 | ns |
|  | Enolase | eno | 46 | 100.00 | 46 | 100.00 | ns |
|  | Cell surface elastin binding protein | ebpS | 46 | 100.00 | 46 | 100.00 | ns |
|  | Fibrinogen binding protein | fib | 43 | 93.48 | 19 | 41.30 | <0.0001 |
|  | Fibronectin-binding protein A | fnbA | 46 | 100.00 | 44 | 95.65 | ns |
|  | MHC Class II Analog protein (=Extracellular adherence protein, eap) | map | 45 | 97.83 | 42 | 91.30 | ns |
|  | *S. aureus* surface protein G | sasG | 44 | 95.65 | 18 | 39.13 | <0.0001 |
|  | Bone Sialoprotein-Binding protein C | sdrC | 46 | 100.00 | 46 | 100.00 | ns |
|  | Van Willebrand Factor - Binding protein | vwb | 45 | 97.83 | 33 | 71.74 | 0.0008 |
|  | Putative transporter protein | ImrP | 46 | 100.00 | 46 | 100.00 | ns |
|  | Immunodominant antigen B | isaB | 46 | 100.00 | 46 | 100.00 | ns |
|  | Transferrin-binding Protein | isdA | 46 | 100.00 | 45 | 97.83 | ns |
|  | Probable lysylphosphatidylglycerol synthetase | mprF | 44 | 95.65 | 31 | 67.39 | 0.0008 |

Statistics was performed by Fisher's exact test. For better readability of the table the genes not detected in the current isolate cohort as well as allelic variants were not shown. Concealed genes and gene components: ermB, mefA, mph(C), vat(A), vat(B), vga, aphA3, sat, dfrS1, far1, cat, fexA, cfr, vanA/B/C, mercury resistance locus, qacA/C, seb,sef, she, seq, PVL, lukM, etB, edinA/ D, splE, vwb, Q2YUB3. Allelic variants of genes: vga, lukF , lukS, lukY, hlIII, aur, map, sdrC, sdrD, vwb, sasG, isaB, mprF, ImrP.
